# Establishment of anti-asialo-GM1 rabbit monoclonal antibodies capable of reducing natural killer cell activity in mice

**DOI:** 10.1371/journal.pone.0292514

**Published:** 2023-10-09

**Authors:** Tatsuji Kimura, Satoshi Ohta, Hiroshi Murayama

**Affiliations:** Diagnostic Division, Yamasa Corporation, Choshi, Chiba, Japan; Tokyo University of Pharmacy and Life Sciences: Tokyo Yakka Daigaku, JAPAN

## Abstract

Rabbit anti-asialo-GM1 (ASGM1) serum or polyclonal antibodies can eliminate mouse splenic natural killer (NK) cell activity in vitro and in vivo. We developed rabbit monoclonal antibodies (mAbs) against ASGM1 using a single-cell analysis and isolation system. Five mAbs (GA109, GA115, GA116, GA131, and GA134) that were reactive to ASGM1 were isolated from the spleen lymphocytes of rabbits immunized with ASGM1. Enzyme-linked immunosorbent assay and thin-layer chromatography immunostaining results showed that the mAbs strongly reacted with ASGM1. Two mAbs (GA116 and GA134) reacted exclusively with ASGM1, whereas three mAbs (GA109, GA115, and GA131) showed slight or considerable cross-reactivity with GM1. The administration of the mAbs (4–20 μg) to BALB/c mice completely abolished NK cell activity in vivo. The anti-ASGM1 rabbit mAbs obtained in this study may provide a useful and reproducible tool for various future studies, such as depleting NK cell activity to enhance xenograft engraftment in mouse models.

## Introduction

Natural killer (NK) cells are effector lymphocytes of the innate immune system that are essential to local and systemic immune surveillance, particularly in eliminating viral-infected and tumor cells. NK cells can lyse several types of tumor cells without prior sensitization [1].

The glycolipid asialo-GM1 (ASGM1, gangliotetraosylceramide), an uncharged analog of GM1 (monosialotetrahexosylganglioside), is expressed on the surface of murine NK cells and is used as an NK cell marker [2, 3]. Rabbit antisera or polyclonal antibodies (PoAbs) against ASGM1 can abolish the NK cell activity of mouse spleen cells and enhance transplanted tumor growth in vivo [2, 4, 5]. These PoAbs are commercially available (Fujifilm Wako Pure Chemical, Osaka, Japan; and BioLegend, San Diego, CA, USA) and widely used in NK cell studies [6, 7]. The elimination of NK cells using anti-ASGM1 PoAbs enhances the engraftment of human cells into immunodeficient mice [5, 8–10]. For example, severe combined immunodeficiency (SCID) mice lack both humoral and cell-mediated immunity owing to the absence of mature B and T lymphocytes and are used as a model animal for implantation of human normal or pathological cells or organs [11]. However, the SCID-mouse NK cells can attack implanted cells. To deplete NK cells and enhance engraftment, SCID mice can be treated with anti-ASGM1 PoAbs prior to the xenograft implantation [8].

PoAbs exhibit lot-to-lot variation, cross-reactivity, and decreased reproducibility. Similarly, anti-ASGM1 PoAbs showed cross-reactivity with GM1, a naturally occurring sialylated derivative of ASGM1, which was somewhat concerning [12, 13]. Nevertheless, anti-ASGM1-mediated NK cell depletion is a powerful tool for analyzing the functions of NK cells.

To resolve these shortcomings, numerous researchers have attempted to obtain monoclonal antibodies (mAbs) against ASGM1 from immunized mice using the conventional hybridoma method [12–15]. However, ASGM1 is expressed on the surface of murine NK cells and other cells, therefore, an autoantigen with low immunogenicity in mice [2]. Some of the reported mAbs exhibited notable or low ability to inactivate NK cells in vitro [14, 15], while others lacked this ability [13]. However, data on in vivo activity of mAbs are lacking, thus, limiting their application.

ASGM1 is highly immunogenic in rabbits [2]. However, there have been no reports on rabbit mAbs against ASGM1. The production of rabbit mAbs has been limited by the technical difficulties associated with the hybridoma-based technology [16]. Over the past three decades, numerous non-hybridoma-based technologies for isolating rabbit mAbs, including display [17] and single B cell antibody technologies [18–20], have been developed. To isolate antigen-specific antibody-secreting cells, Jin et al. developed a technology using a microwell alley chip called immunospot array assay on a chip (ISAAC) [18, 21]. Furthermore, the automated single-cell analysis and isolation system is an efficient method for the rapid screening and isolation of antibody-secreting cells [22]. A single lymphocyte secreting the desired antibody can be isolated by detecting the target cells on a microchamber array chip. By combining these technologies, mAbs can efficiently be obtained from rabbits and other animals.

In this study, we aimed to generate rabbit mAbs against ASGM1 using an automated single-cell picking system. We successfully generated and characterized five mAbs against ASGM1. Two of the five mAbs reacted exclusively with ASGM1, whereas three showed considerable or slight cross-reactivity with GM1. All five mAbs abolished NK cell activity in mouse spleen cells in vivo. These mAbs against ASGM1 provide specific and stable reagents for the study of murine NK cells.

## Materials and methods

### Animals

Rabbit and mouse experiments were approved by the Committee for Animal Experiments of the Yamasa Corporation (21-S-10, 21-S-11, 22-S-01, 22-S-02, and 23-S-05).

Japanese white female rabbits were supplied by Shiraishi Laboratory Animals (Koshigaya, Japan). Female BALB/c mice were obtained from Japan SLC (Hamamatsu, Japan) and used at 6–8 weeks of age.

### Glycolipids

ASGM1 for immunization was purchased from Trina Bioriactives (Naenikon, Switzerland). For enzyme-linked immunosorbent assay (ELISA) and thin-layer chromatography (TLC), GM1, GM2, GM3, GD1a, GD1b, ASGM1, and Asialo-GM2 (ASGM2) were purchased from Adipogen (San Diego, CA, USA), and lactosylceramide (Asialo-GM3, ASGM3) from Cayman Chemical (Ann Arbor, MI, USA).

The structures of glycolipids are presented in Table 1.

**Table 1:** Structures of glycolipids.

Name	Structure
GM1	Gal(β1–3)GalNAc(β1–4)[NeuAc(α2–3)]Gal(β1–4)Glc(β1–1)Cer^a^
GM2	GalNac(β1–4)[NeuAc(α2–3)]Gal(β1–4)Glc(β1–1)Cer
GM3	NeuAc(α2–3)Gal(β1–4)Glc(β1–1)Cer
GD1a	NeuAc(α2–3)Gal(β1–3)GalNAc(β1–4)[NeuAc(α2–3)]Gal(β1–4)Glc(β1–1)Cer
GD1b	Gal(β1–3)GalNAc(β1–4)[NeuAc(α2–8)NeuAc(α2–3)]Gal(β1–4)Glc(β1–1)Cer
Asialo GM1	Gal(β1–3)GalNac(β1–4)Gal(β1–4)Glc(β1–1)Cer
Asialo GM2	GalNac(β1–4)Gal(β1–4)Glc(β1–1)Cer
Asialo GM3	Gal(β1–4)Glc(β1–1)Cer

^a^ Ceramide, N-acylsphingosine

Abbreviations: GM1: monosialotetrahexosylganglioside; GM2: monosialoganglioside GM2; GM3: monosialodihexosylganglioside; GD1a: disialoganglioside GD1a; GD1b: disialoganglioside GD1b; asialo GM1: gangliotetraosylceramide, GA1; asialo GM2: gangliotriaosylceramide, GA2; asialo GM3: lactosylceramide.

### Production of anti-asialo-GM1 rabbit monoclonal antibodies

Liposomes containing ASGM1 (Trina) and bovine serum albumin (BSA) were prepared, suspended in phosphate-buffered saline (PBS), and emulsified with the same volume of Freund’s adjuvant. Six rabbits were used for immunization because the antibody titers exhibited large individual differences. Japanese white rabbits were immunized subcutaneously with 2 mg/0.5 mL ASGM1 emulsion with Freund’s complete adjuvant. At 2 and 4 weeks after the primary immunization, the rabbits were boosted subcutaneously with 1 mg/0.5 mL ASGM1 emulsion with Freund’s incomplete adjuvant. Ten to twelve days after the third immunization, they were euthanized under general anesthesia using ketamine hydrochloride and xylazine hydrochloride injection. Spleen cells were collected from the rabbit and erythrocytes were lysed in a hypotonic solution (red blood cell lysing buffer; Sigma-Aldrich, Merck, Darmstadt, Germany). The lymphocytes obtained were then aliquoted and stocked at -80°C until application. An aliquot of splenic lymphocytes from a rabbit that exhibited high anti-ASGM1 secretion capability was selected, and a different aliquot from the same rabbit was used for mAb isolation.

Rabbit mAbs were produced by the Cell Engineering Corporation (Osaka, Japan) using an automated single-cell analysis and isolation system [22], which enabled high-throughput screening based on a microchamber array chip single picking system (AS ONE, Osaka, Japan). The prepared spleen lymphocytes (2 x 10^5^ cells, 4 times) were introduced into microchambers (10 μm diameter, 256,000 microwell array on a chip) that were pre-coated with 3 μg/mL ASGM1 antigen. The cells in each microchamber were evaluated for their anti-ASGM1 antibody-secreting ability using an on-chip fluorescence immunoassay with an Alexa Fluor 488-conjugated goat anti-rabbit Immunoglobulin G (IgG). The fluorescence intensity of each microchamber on the chip was measured. Anti-ASGM1 positive B-cells were selected using a glass capillary attached to an automated micromanipulator. RNA was extracted from the cells, and antibody (VH, VL) cDNA was amplified using reverse transcription polymerase chain reaction (RT-PCR). Human embryonic kidney (HEK) 293 cells were transfected with cDNAs encoding the heavy and light chains. The antibodies secreted into the culture medium were screened through ELISA (described below). Five anti-ASGM1-positive clones were selected, and the cDNA of the heavy and light chains were inserted into the expression vector (Cell Engineering Corporation). The reactivity of the antibodies in the culture supernatant of HEK293 cells transfected with the vector was confirmed using ASGM1-coated ELISA. Recombinant mAbs were expressed in HEK293 cells cultured in HE-200 serum-free medium (Gmep Incorporated, Kurume, Fukuoka, Japan) and purified from the culture supernatant using protein A (Cytiva, Tokyo, Japan) affinity chromatography.

### Other natural killer-depleting antibodies

Two NK-depleting antibodies were used as controls. The concentration of rabbit anti-ASGM1 PoAb (Fujifilm Wako Pure Chemical, Osaka, Japan) is not regulated, thus the quantities are expressed in μL. TM-β1, a mAb against murine interleukin-2 receptor β chain (IL-2Rβ, CD122), was purchased from Immuno-Biological Laboratories (IBL, Fujioka, Japan).

### Glycolipid-coated enzyme-linked immunosorbent assay

Antibody titers against ASGM1 or other glycolipids were measured using ELISA. The 96-well ELISA plates (Iwaki, AGC Techno Glass, Shizuoka, Japan) were treated with 50 μL ASGM1 per well or structurally related glycolipid (Table 1) solution (1 μg/mL) in ethanol or ethanol-chloroform and then vacuum dried. The wells were blocked with phosphate-buffered saline (PBS) containing 1% gelatin (Sigma-Aldrich, St. Louis, MO, USA) and 1 mM ethylenediamine-N,N,N’,N’-tetraacetic acid (EDTA). After the washout of blocking solution, 100 μL samples were applied to the wells, and incubated for 1 h at room temperature. The samples were then washed thrice with PBS containing 0.05% Tween 20 (T-PBS), followed by the addition of horseradish peroxidase (HRP)-conjugated anti-rabbit IgGs (The Jackson Laboratory, Bar Harbor, ME, USA) and incubation for 1 h. After the washout of free conjugate, 100 μL 3,3,5,5,-tetramethylbenzidine (TMB) substrate was added and incubated for 30 min; the reaction was stopped with 50 μL 2 M H_2_SO_4_. Absorbance at 450 nm was measured using a microplate reader (Tecan, Männedorf, Switzerland).

### Sandwich enzyme-linked immunosorbent assay for whole rabbit immunoglobulin G

Rabbit IgG concentration was measured by sandwich ELISA, using the aforementioned procedure, except that the ELISA wells were coated with anti-rabbit IgG antibodies instead of glycolipid antigens, i.e., 96-well ELISA plates were treated with 100 μL anti-rabbit IgG (1 μg/mL in PBS; The Jackson Laboratory, Bar Harbor, ME, USA) per well.

### Thin-layer chromatography immunostaining

Immunostaining of glycolipids on TLC sheets was performed according to the method described by Watarai et al. [23], with certain modifications. Glycolipids (1 μg) were spotted onto TLC silica gel polyester sheets (100 × 100 mm, Polygram, Macherey-Nagel, Germany) and developed with chloroform/methanol/water/acetic acid (60:40:6:0.2 vol.) at room temperature for 30 min and then air-dried. Glycolipids on the TLC sheets were visualized with a sprayed 5-methylresorcinol (Orcinol, Tokyo Chemical Industry, Tokyo, Japan) solution (2 mg/mL in 2 M H_2_SO_4_) and heated at 110°C for 5 min. For immunostaining, TLC sheets were immersed in PBS containing 1% gelatin, 1% polyvinylpyrrolidone (PVP-K30, Nacalai Tesque, Kyoto, Japan), and 1 mM EDTA, followed by immersion in anti-ASGM1 rabbit mAbs (50 ng/mL) or PoAb (1:1,000) solution and allowed to react for 1 h at room temperature. After washing thrice with T-PBS, HRP-conjugated anti-rabbit IgG (1:40,000; The Jackson Laboratory, Bar Harbor, ME, USA) was added. After washing thrice with T-PBS, the TLC sheets were incubated in TMB solution for western blotting (Nacalai Tesque, Kyoto, Japan). Consequently, the bound antibody was visualized in blue.

### Cytotoxicity assay

Antibodies (0, 0.8, 4, 20, 100 μg/head) were intraperitoneally injected into female BALB/c mice (n = 2). Two days later, 200 μg polyinosinic-polycytidylic acid (Poly I:C) (Yamasa Corporation, Chiba, Japan), a double-stranded RNA analog, was intraperitoneally injected into the mice to activate NK cells [24]. On day 3, they were euthanized by cervical dislocation. Spleen cells were harvested from the mice, and the erythrocytes were lysed in a hypotonic solution (Red Blood Cell Lysing Buffer; Sigma-Aldrich, Merck, Darmstadt, Germany).

NK cell-mediated cytotoxicity was determined by measuring lactate dehydrogenase (LDH) released from target cells [25]. The Moloney virus-induced lymphoma cell line YAC-1 was used as the target cell for NK activity. Freshly prepared splenic lymphocytes (effector: 1 × 10^6^ or 5 × 10^5^ cells/well) were incubated with YAC-1 target cells (2 × 10^4^ cells/well) (effector:target = 50:1 or 25:1) quadrupled in RPMI (Gibco, Thermo Fisher Scientific, Waltham, MA, USA) supplemented with 0.6% BSA on a flat-bottom 96-well cell culture plate (Iwaki, AGC Techno Glass, Shizuoka, Japan) for 4 h at 37°C in 5% CO_2_. LDH activity in the culture supernatant was measured using the CytoTox 96 Non-Radioactive Cytotoxicity Assay Kit (Promega, Madison, WI, USA). Cytotoxicity was expressed as % target lysis according to the following formula:

% Cytotoxicity = (Experimental Response—Effector Spontaneous Response—Target Spontaneous Response) / (Target Maximum Response—Target Spontaneous Response) × 100, where Experimental Response is the mean LDH activity in the supernatant of effector and target coculture wells, Effector Spontaneous Response is that of effector-only wells, Target Spontaneous Response is that of target-only wells, and Target Maximum Response is that of target-only wells with detergent addition to lyse all target cells.

## Results

### Isolation of anti-asialo-GM1 rabbit monoclonal antibodies

Splenic lymphocytes were obtained from six rabbits immunized thrice with ASGM1 antigen. Lymphocytes from a rabbit that secreted high titers of anti-ASGM1 were selected and used for mAb isolation. Forty anti-ASGM1-positive clones were isolated from the spleen cells using a single-cell picking system. Forty-pair fragments of the variable region sequence, heavy and light chains, were amplified using single-cell RT-PCR. The antibodies expressed in HEK293 cells transfected with cDNA were evaluated by ELISA for anti-ASGM1 and whole-rabbit IgG (S1 Table). Antibody affinities were estimated using anti-ASGM1/rabbit IgG ratio. Among the 40 clones, 22 mAbs significantly reacted with ASGM1 in ELISA. We selected five clones of anti-ASGM1, expecting a high affinity for three of them and different properties for two of them (S1 Table). Each cDNA set was inserted into the expression vector and HEK293 cells were transfected. Reactivity of the antibodies in the culture supernatant of HEK293 cells was confirmed. Finally, we established five clones (GA109, GA115, GA116, GA131, and GA134) of rabbit IgG mAbs reactive to ASGM1.

Analysis of the mAb sequences showed that all the antibodies had unique nucleotide and deduced amino acid sequences.

### Reactivity and specificity on glycolipid-coated enzyme-linked immunosorbent assay

**Fig 1:**
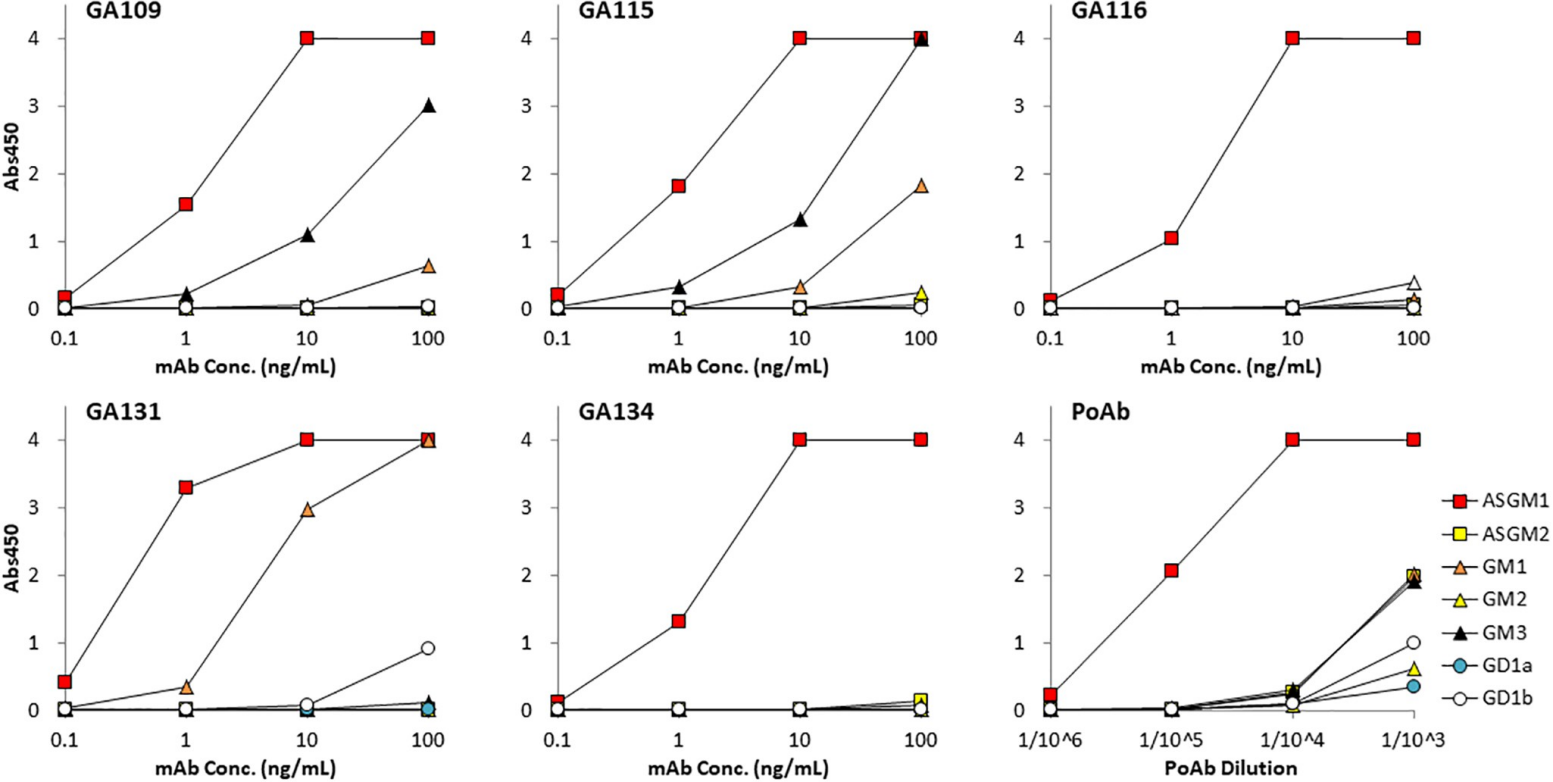
Specificity of anti-ASGM1 antibody on ELISA. Serially diluted anti-ASGM1 mAbs (GA109, GA115, GA116, GA131, and GA134) or PoAb were added to wells coated with glycolipids (ASGM1, ASGM2, GM1, GM2, GM3, GD1a, and GD1b). Antibodies were detected using anti-rabbit IgG-HRP/TMB. The ELISA results are indicated as absorbance at 450 nm. Graphs show representative data from at least three independent single-well experiments. ELISA, enzyme-linked immunosorbent assay.

We examined the reactivity and specificity of the mAbs by ELISA using ASGM1 or structurally related glycolipids as antigens (Table 1, Fig 1). The control PoAb exhibited strong reactivity with ASGM1 and low cross-reactivity with ASGM2, GM1, and GM3. All five mAbs exhibited strong reactivity against ASGM1, whereas no binding was observed for ASGM2, GM2, or GD1a. Cross-reactivity with GM1 can be classified into three groups: group 1, GA116 and GA134, represented no reactivity; group 2, GA109 and GA115, represented slight reactivity; and group 3, GA131, represented considerable reactivity. The GA131 mAb showed slight cross-reactivity with GD1b, whereas the other four mAbs showed none. Furthermore, GA109 and GA115 displayed reactivity toward the GM3 coat.

GM1 and GD1b have molecular similarities to ASGM1, but their GM3 structures are completely different from that of ASGM1 (Table 1). The reasons underlying the reaction of the anti-ASGM1 antibody with GM3 were investigated.

### Reactivity and specificity on thin-layer chromatography immunostaining

**Fig 2:**
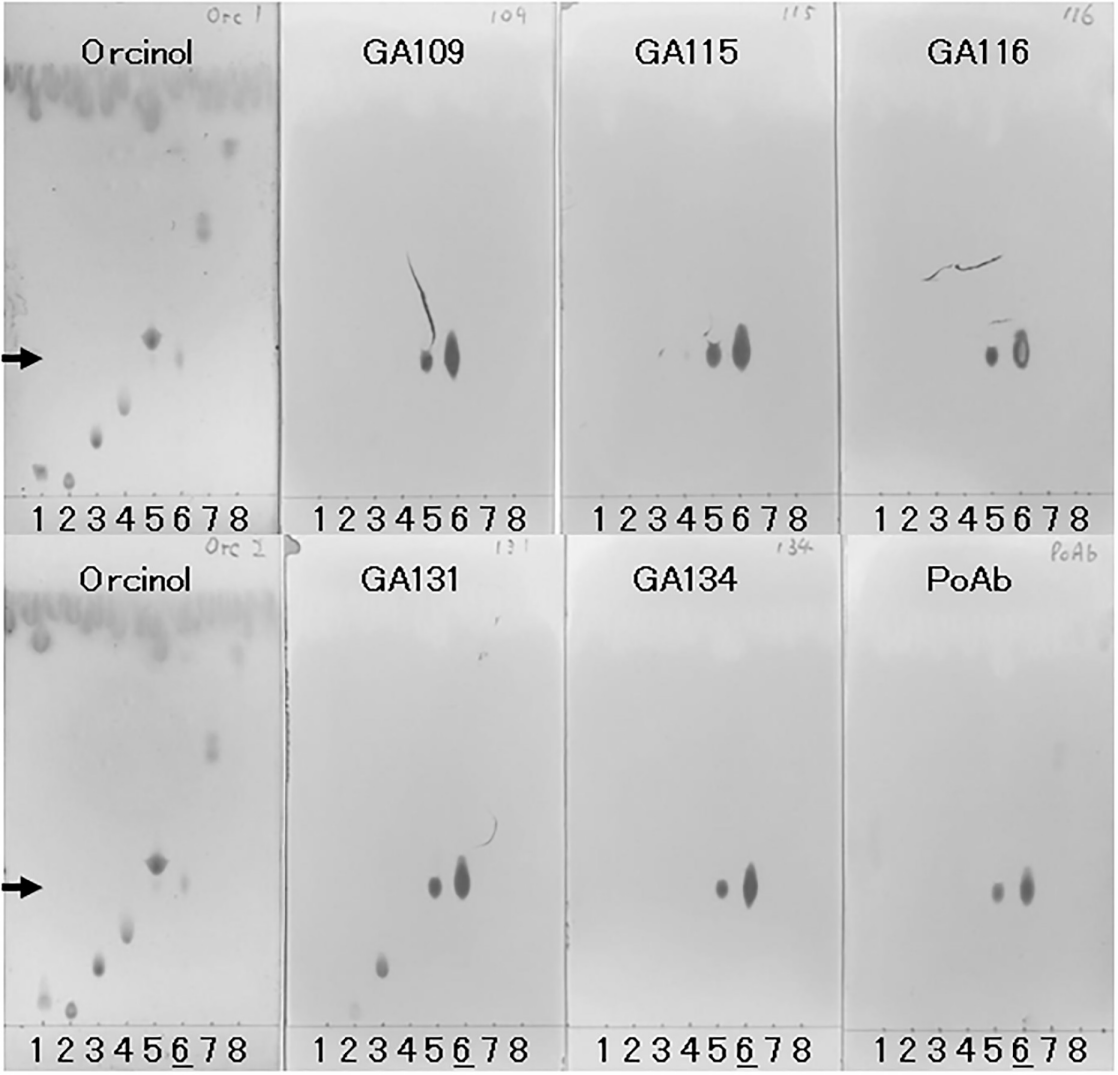
Specificity of anti-ASGM1 antibody on TLC-Immunostaining. Glycolipids were developed through silica gel TLC. Anti-ASGM1 mAbs (GA109, GA115, GA116, GA131, and GA134) or PoAb bound to the TLC sheet were detected using anti-rabbit IgG-HRP/TMB. Glycolipids on the TLC sheets were visualized using Orcinol. The glycolipids were organized according to lane as follows: 1, GD1a; 2, GD1b; 3, GM1; 4, GM2; 5, GM3; 6, ASGM1; 7, ASGM2; 8, Lactosylceramide (ASGM3). Arrowhead points to the ASGM1 migration. TLC, thin-layer chromatography; mAb, monoclonal antibody; PoAb, polyclonal antibody.

We further examined the reactivity of the mAbs by TLC-immunostaining (Fig 2). ASGM1 and structurally related glycolipids (GD1a, GD1b, GM1, GM2, GM3, ASGM2, and ASGM3; Table 1) were spotted and developed by silica-gel TLC. One set of developed TLC sheets was visualized using Orcinol staining (Fig 2, Orcinol). Each glycolipid was separated by the TLC. ASGM1 (lane 6) evidently migrated less than GM3 (lane 5). Other sets of TLC sheets that were developed simultaneously were immunostained with anti-ASGM1 antibodies. All mAbs (GA109, GA115, GA116, GA131, and GA134) and PoAb reacted with ASGM1 spots (lane 6). In addition, all mAbs and PoAb exhibited a relatively small spot on each GM3 lane (lane 5) that migrated the same distance as ASGM1 (arrowhead), which differed from the upper GM3 main spot (Orcinol, lane 5). These data indicate that mAbs react with small amounts of impurities (probably ASGM1) in the GM3 sample, but do not react with GM3.

These results (Figs 1 and 2) show that the specificities of the mAbs are as follows: group 1 mAbs (GA116 and GA134) react with ASGM1 but not with the other glycolipids tested, group 2 mAbs (GA115 and GA109) exhibit slight cross-reactivity with GM1, while group 3 mAb (GA131) cross-react considerably with GM1 and slightly with GD1b.

### Reduction of natural killer cell activity of mice in vivo

**Fig 3:**
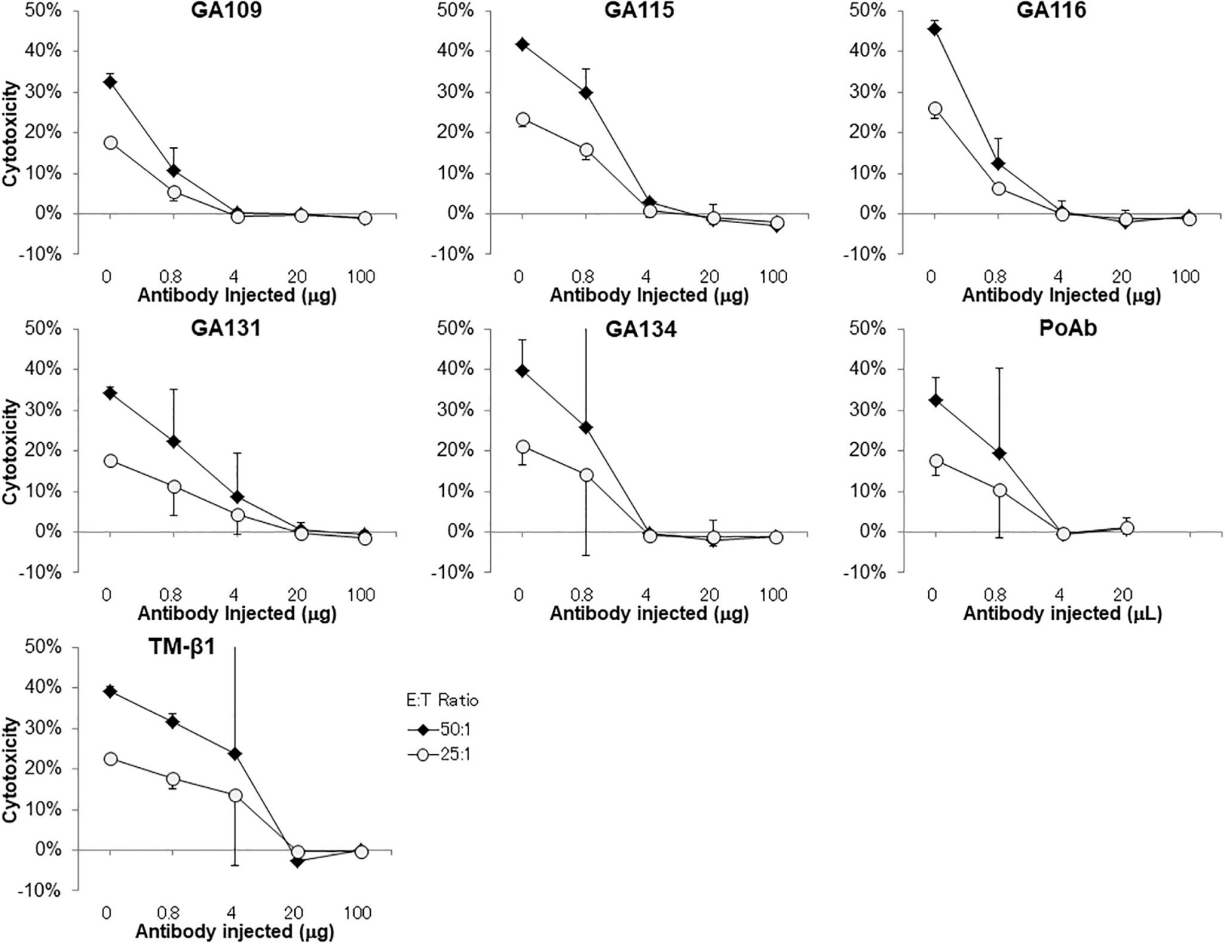
NK cell activity of spleen lymphocytes from antibody injected BALB/c mice. YAC-1 target cells were incubated with effector splenic lymphocytes from poly I:C-stimulated BALB/c mice (n = 2) 3 days after antibody injection. Cytotoxicity against YAC-1 cells was measured using an LDH release assay at effector:target (E:T) ratios of 50:1 and 25:1. The percentages of specific cell lysis are indicated as cytotoxicity. Data are presented as mean ± SD of two mice. LDH, lactate dehydrogenase; SD, standard deviation; NK, natural killer.

We examined the effect of in vivo administration of anti-ASGM1 mAbs for NK depletion in poly I:C stimulated mice and compared it with that of NK cell depleting anti-ASGM1 PoAb or anti-IL-2Rβ mAb TM-β1. BALB/c mice were injected with varying doses of the anti-ASGM1 mAbs, PoAb, or TM-β1 mAb. Three days later, NK cell activity against YAC-1 in the splenic lymphocytes of antibody-treated mice was determined using an LDH release assay (Fig 3). NK activity was completely abolished with >4 μg of GA109, GA115, GA116, and GA134 mAbs, >20 μg of GA131 and TM-β1 mAbs, or >4 μL of anti-ASGM1 PoAb injection. All five mAbs against ASGM1 exhibited strong ability to reduce NK cell activity, similar to anti-ASGM1 PoAb or TM-β1.

## Discussion

Since the discovery of its in vivo effect in 1981 [4], anti-ASGM1 rabbit sera or PoAbs have been used for NK cell depletion in mice [5, 8, 9]. Although PoAbs are useful reagents, mAbs are preferred as they are more refined and consistent reagents. During 1987 to 1988, four studies successively reported murine IgM mAbs against ASGM1 [12–15]. Certain mAbs have been reported to deplete NK cells treated with antibodies in vitro [14, 15]; however, no mAb has been reported to be effective when injected into mice (i.e., in vivo). Consequently, these mAbs have not been as widely used as PoAbs. ASGM1 is an autoantigen in mice but is highly immunogenic in rabbits. We expected that if rabbit mAbs with strong reactivity to ASGM1 were to be produced using recent technologies, a few of the mAbs may exhibit NK depletion activity in vivo.

Rabbit mAbs generally exhibit high affinity and specificity even for low-immunogenic antigens in mice [16, 26, 27]. However, the production of rabbit mAbs has been hampered by technical complications. Hybridoma-based technology for producing rabbit mAbs has low cell fusion efficiency, whereas the phage display method results in the loss of natural cognate pairing of heavy and light chains [16, 27]. Single B cell antibody technology can resolve these issues. Single B-cell-based technology circumvents the inefficient hybridoma fusion step and retains natural heavy- and light-chain pairing.

In this study, we successfully obtained five clones of rabbit mAbs against ASGM1 from spleen lymphocytes of ASGM1 immunized rabbit, using an automated single-cell isolation system [22]. All five mAbs were highly effective, as assessed by the abolishment of NK activity in mice in vivo. The cytolytic activity against NK-sensitive YAC-1 in the spleen lymphocytes of antibody-injected BALB/c mice was completely depleted (Fig 3). To the best of our knowledge, this is the first report on anti-ASGM1 mAbs capable of abolishing mouse NK activity in vivo.

Numerous mice are required in the study of the in vivo effects of mAbs. For example, Fig 3 represents the results from 68 heads of mice. Nevertheless, the results were limited to two heads of mice per condition. In addition, the NK activity was observed at a single point, i.e., at 3 days after administration of mAb. Further studies on a large number of mice or with innovative methods are needed for detailed evaluation.

Immunocompromised mice such as nude, SCID, or non-obese diabetic (NOD)/SCID mice retain intact or impaired NK cell functions that hinder the engraftment of transplanted xenograft. More combined immunocompromised mice lacking NK cells such as NOD/Shi-scid IL-2Rγ^null^ (NOG, NSG) mice have been developed and show relatively high engraftment efficiencies in patient-derived xenograft (PDX) models [28]. However, highly immunocompromised mice require especially clean conditions, and cannot breed by users; making their maintenance costly. Conversely, the maintenance of nude, SCID, or NOD/SCID mice is relatively easy and cost-effective, still making them important resources for PDX establishment. The PoAb against ASGM1 have been used to deplete NK cells and increase engraftment rate. Similarly, mAbs in the present study would be beneficial for efficient engraftment in these mice.

We evaluated the mAbs for reactivity to ASGM1 and cross-reactivity to related glycolipids using ELISA and TLC-immunostaining (Figs 1 and 2). The five mAbs can be categorized into three groups: group 1, GA116 and GA134; group 2, GA109 and GA115; and group 3, GA131. Both GA109 and GA115 mAbs, which initially indicated relatively low estimated affinity and were expected to differ from others (S1 Table), belonged to group 2 and exhibited ASGM1-binding ability exceeding that of group 1 mAbs (GA116 and GA134) (Fig 1). The group orders of reactivity and specificity to ASGM1 were 3 > 2 > 1 and 1 > 2 > 3, respectively, implying an inverse relationship. GA131 mAb showed the strongest cross-reactivity to GM1 (Figs 1 and 2) and required the highest dose to deplete NK activity in vivo (Fig 3). There may be a relationship between cross-reactivity and the minimum dose required for NK cell depletion.

The TLC-immunostaining results implied that the GM3 sample contained ASGM1 as an impurity, which was recognized by all five clones of anti-ASGM1 mAbs and PoAb (Fig 2). In the antigen-coated ELISA, GA109 and GA115 mAbs exhibited reactivity with the GM3 coat, whereas GA116, GA131, and GA134 mAbs did not (Fig 1). We assume the reasons underlying this discrepancy as follows. All five mAbs reacted with the ASGM1 spot on the TLC gel separated from the GM3 sample, which contained ASGM1 as an impurity. In contrast, for the antigen-coated ELISA, considerably less ASGM1 was attached to the surface of GM3 treated wells, which was almost exclusively coated with GM3. Consequently, the reaction to a subtle amount of ASGM1 on GM3 wells was detected for certain mAbs (GA115 and GA109), but not for others (GA116, GA131, and GA134).

Anti-ASGM1 depletion is not only restricted to NK cells but also to cytotoxic T lymphocytes [29, 30], macrophages [14], or basophils [31]. However, anti-ASGM1 PoAbs cross-react with GM1 [12, 13]. Our results indicated that the PoAb cross-reacted with ASGM2 in addition to GM1 (Figs 1 and 2). Whether the effects on non-NK cells are due to the real responses of anti-ASGM1 or the cross-reactivity of the PoAbs remains unclear. Our mAbs for ASGM1, particularly ASGM1-specific clones (GA116 and GA134), are critical tools for elucidating these issues.

PoAbs against ASGM1 have been widely used in NK cell suppression studies. Alternatively, injection of the TM-β1, a rat mAb against murine IL-2Rβ, into mice resulted in elimination of splenic NK function in vivo [30, 32, 33]. Typically, 100–1,000 μg of TM-β1 were administered to mice [33, 34]. By contrast, a 20 μg dose of TM-β1 injection was sufficient to inhibit NK cell activity (Fig 3). Moreover, lower dose (4 μg) of anti-ASGM1 mAb (GA109, GA115, GA116, or GA134) was sufficient to completely deplete NK cell activity. However, the significance of the variation in the minimum dose was unclear in this study. In addition, we did not study the duration of NK cell activity by the rabbit mAbs; moreover, rat mAb TM-β1 was reported to last longer (up to 5–7 weeks) than the anti-ASGM1 rabbit PoAbs [5, 10, 32]. The dose and/or species of mAbs injected into the mice could be related to the duration of efficacy. The recombinant mAb reformatted to mouse IgG was expected to be more effective and last longer. In addition, difference in the effectivity of mouse IgG subtypes (IgG1, IgG2a IgG2b, or IgG3) need to be investigated. These issues should be addressed in future studies.

Anti-NK1.1 mAb also exhibits NK depletion activity [35]. However, anti-NK1.1-mediated NK cell depletion works only in certain strains, such as C57BL/6 and SJL, and does not work in numerous other strains lacking the NK1.1 allotype, including BALB/c, C3H, and A/J mice [36]. The anti-ASGM1-mediated NK cell depletion was effective in various mouse strains [30]. NK cell inactivation by anti-ASGM1 mAbs was expected to be effective regardless of the mouse strain used.

We developed rabbit mAbs against ASGM1 that can eliminate NK cell activity in mice in vivo. These mAbs could provide an alternative tool for NK cell studies or for depleting NK activity in mouse models.

## Supporting information

S1 Table Antibody titer in transfected HEK293 cell culture supernatant. Antibodies in serially diluted culture supernatants of HEK293 cells transfected with the antibody cDNA were measured using ELISA for anti-ASGM1 and whole-rabbit IgG. The results were reorganized according to the anti-ASGM1 titer order. The anti-ASGM1/rabbit IgG ratios were calculated from the absorbance at the lowest dilution within a linear range (Absorbance < 2.0 anti-ASGM1 ELISA).
(TIF)

S1 Raw images Raw images of Fig 2. (PDF)

## References

[1] Vivier E, Tomasello E, Baratin M, Walzer T, Ugolini S. Functions of natural killer cells. Nat Immunol. 2008;9: 503–510. doi: 10.1038/ni1582 18425107

[2] Kasai M, Iwamori M, Nagai Y, Okumura K, Tada T. A glycolipid on the surface of mouse natural killer cells. Eur J Immunol. 1980;10: 175–180. doi: 10.1002/eji.1830100304 6966574

[3] Young WW Jr, Hakomori SI, Durdik JM, Henney CS. Identification of ganglio-N-tetraosylceramide as a new cell surface marker for murine natural killer (NK) cells. J Immunol. 1980;124: 199–201. 6985637

[4] Kasai M, Yoneda T, Habu S, Maruyama Y, Okumura K, Tokunaga T. In vivo effect of anti-asialo GM1 antibody on natural killer activity. Nature 1981;291: 334–335. doi: 10.1038/291334a0 7231554

[5] Habu S, Fukui H, Shimamura K, Kasai M, Nagai Y, Okumura K, et al. In vivo effects of anti-asialo GM1. I. Reduction of NK activity and enhancement of transplanted tumor growth in nude mice. J Immunol. 1981;127: 34–38. 7240748

[6] Victorino F, Sojka DK, Brodsky KS, McNamee EN, Masterson JC, Homann D, et al. Tissue-resident NK cells mediate ischemic kidney injury and are not depleted by anti-asialo-GM1 antibody. J Immunol. 2015;195: 4973–4985. doi: 10.4049/jimmunol.1500651 26453755 PMC4640895

[7] Monnier J, Zabel BA. Anti-asialo GM1 NK cell depleting antibody does not alter the development of bleomycin induced pulmonary fibrosis. PLoS One. 2014;9: e99350. doi: 10.1371/journal.pone.0099350 24922516 PMC4055641

[8] Barry TS, Jones DM, Richter CB, Haynes BF. Successful engraftment of human postnatal thymus in severe combined immune deficient (SCID) mice: differential engraftment of thymic components with irradiation versus anti-asialo GM-1 immunosuppressive regimens. J Exp Med. 1991;173: 167–180. doi: 10.1084/jem.173.1.167 1985120 PMC2118746

[9] Yoshino H, Ueda T, Kawahata M, Kobayashi K, Ebihara Y, Manabe A. Natural killer cell depletion by anti-asialo GM1 antiserum treatment enhances human hematopoietic stem cell engraftment in NOD/Shi-scid mice. Bone Marrow Transplant. 2000;26: 1211–1216. doi: 10.1038/sj.bmt.1702702 11149733

[10] Yano S, Nishioka Y, Izumi K, Tsuruo T, Tanaka T, Miyasaka M, et al. Novel metastasis model of human lung cancer in SCID mice depleted of NK cells. Int J Cancer. 1996;67: 211–217. doi: 10.1002/(SICI)1097-0215(19960717)67:2&lt;211::AID-IJC11&gt;3.0.CO;2-E 8760590

[11] Bosma MJ, Carroll AM. The SCID mouse mutant: definition, characterization, and potential uses. Annu Rev Immunol. 1991;9: 323–350. doi: 10.1146/annurev.iy.09.040191.001543 1910681

[12] Jacquemart F, Millot G, Goujet-Zalc C, Mahouy G, Zalc B. Production and characterization of a mouse monoclonal antibody to the glycolipid asialo-GM1. Hybridoma. 1988;7: 323–331. doi: 10.1089/hyb.1988.7.323 3169804

[13] Watarai S, Handa S, Tadakuma T, Yasuda T. Application of liposomes to generation of monoclonal antibody to glycosphingolipid: production of monoclonal antibody to GgOse4Cer. J. Biochem. 1987;102: 59–67. doi: 10.1093/oxfordjournals.jbchem.a122041 3667566

[14] Solomon FR, Higgins TJ. A monoclonal antibody with reactivity to asialo GM1 and murine natural killer cells. Mol Immunol. 1987;24: 57–65. doi: 10.1016/0161-5890(87)90111-8 3614206

[15] Shimada S, Iwata D. Preparation of monoclonal antibodies against a glycolipid asialo GM1. Microbiol Immunol. 1987;31: 923–933. doi: 10.1111/j.1348-0421.1987.tb03153.x 3696009

[16] Weber Justus, Peng Haiyong, Rader Christoph. From rabbit antibody repertoires to rabbit monoclonal antibodies. Exp Mol Med. 2017;49: e305. doi: 10.1038/emm.2017.23 28336958 PMC5382564

[17] Ridder R, Schmitz R, Legay F, Gram H. Generation of rabbit monoclonal antibody fragments from a combinatorial phage display library and their production in the yeast Pichia pastoris. Biotechnology. 1995;13: 255–260. doi: 10.1038/nbt0395-255 9634767

[18] Ozawa T, Piao X, Kobayashi E, Zhou Y, Sakurai H, Andoh T, et al. A novel rabbit immunospot array assay on a chip allows for the rapid generation of rabbit monoclonal antibodies with high affinity. PLoS One. 2012;7: e52383. doi: 10.1371/journal.pone.0052383 23300658 PMC3530603

[19] Seeber S, Ros F, Thorey I, Tiefenthaler G, Kaluza K, Lifke V, et al. A robust high throughput platform to generate functional recombinant monoclonal antibodies using rabbit B cells from peripheral blood. PLoS One. 2014;9: e86184. doi: 10.1371/journal.pone.0086184 24503933 PMC3913575

[20] Clargo AM, Hudson AR, Ndlovu W, Wootton RJ, Cremin LA, O’Dowd VL, et al. The rapid generation of recombinant functional monoclonal antibodies from individual, antigen-specific bone marrow-derived plasma cells isolated using a novel fluorescence-based method. MAbs. 2014:6: 143–159. doi: 10.4161/mabs.27044 24423622 PMC3929438

[21] Jin A, Ozawa T, Tajiri K, Obata T, Kondo S, Kinoshita K, et al. A rapid and efficient single-cell manipulation method for screening antigen-specific antibody-secreting cells from human peripheral blood. Nat Med. 2009;15: 1088–1092. doi: 10.1038/nm.1966 19684583

[22] Yoshimoto N, Kida A, Jie X, Kurokawa M, Iijima M, Niimi T. An automated system for high-throughput single cell-based breeding. Sci Rep. 2013;3: 1191. doi: 10.1038/srep01191 23378922 PMC3561619

[23] Watarai S, Kiura K, Shigeto R, Shibayama T, Kimura I, Yasuda T. Establishment of monoclonal antibodies specific for ganglioside GM1: detection of ganglioside GM1 in small cell lung carcinoma cell lines and tissues. J Biochem. 1994;116: 948–954. doi: 10.1093/oxfordjournals.jbchem.a124651 7896755

[24] Cohen SA, Tzung SP, Doerr RJ, Goldrosen MH, Role of asialo-GM1 positive liver cells from athymic nude or polyinosinic-polycytidylic acid-treated mice in suppressing colon-derived experimental hepatic metastasis. Cancer Res. 1990;50: 1834–1840. 2306736

[25] Decker T, Lohmann-Matthes ML. A quick and simple method for the quantitation of lactate dehydrogenase release in measurements of cellular cytotoxicity and tumor necrosis factor (TNF) activity. J Immunol Methods. 1988;115: 61–69. doi: 10.1016/0022-1759(88)90310-9 3192948

[26] Rossi S, Laurino L, Furlanetto A, Chinellato S, Orvieto E, Canal F, et al. Rabbit monoclonal antibodies: A comparative study between a novel category of immunoreagents and the corresponding mouse monoclonal antibodies. Am J Clin Pathol. 2005;124: 295–302. doi: 10.1309/NR8H-N08G-DPVE-MU08 16040303

[27] Zhang Z, Liu H, Guan Q, Wang L, Yuan H. Advances in the isolation of specific monoclonal rabbit antibodies. Front Immunol. 2017;8: 494. doi: 10.3389/fimmu.2017.00494 28529510 PMC5418221

[28] Okada S, Vaeteewoottacharn K, Kariya R. Application of highly immunocompromised mice for the establishment of patient-derived xenograft (PDX) Models. Cells. 2019;8: 889. doi: 10.3390/cells8080889 31412684 PMC6721637

[29] Stitz L, Baenziger J, Pircher H, Hengartner H, Zinkernagel RM. Effect of rabbit anti-asialo GM1 treatment in vivo or with anti-asialo GM1 plus complement in vitro on cytotoxic T cell activities. J Immunol. 1986;136: 4674–4680. 3486908

[30] Ehl S, Nuesch R, Tanaka T, Myasaka M, Hengartner H, Zinkernagel R. A comparison of efficacy and specificity of three NK depleting antibodies. J Immunol Methods. 1996;199: 149–153. doi: 10.1016/s0022-1759(96)00175-5 8982356

[31] Nishikado H, Mukai K, Kawano Y, Minegishi Y, Karasuyama H. NK cell-depleting anti-asialo GM1 antibody exhibits a lethal off-target effect on basophils in vivo. J Immunol. 2011;186: 5766–5771. doi: 10.4049/jimmunol.1100370 21490162

[32] Tanaka T, Kitamura F, Nagasaka Y, Kuida K, Suwa H, Miyasaka M. Selective long-term elimination of natural killer cells in vivo by an anti-interleukin 2 receptor beta chain monoclonal antibody in mice. J Exp Med. 1993;178: 1103–1107. doi: 10.1084/jem.178.3.1103 8350049 PMC2191182

[33] Tournoy KG, Depraetere S, Meuleman P, Leroux-Roels G, Pauwels RA. Murine IL-2 receptor beta chain blockade improves human leukocyte engraftment in SCID mice. Eur J Immunol. 1998;28: 3221–3230. doi: 10.1002/(SICI)1521-4141(199810)28:10&lt;3221::AID-IMMU3221&gt;3.0.CO;2-S 9808191

[34] Yokoyama S, Watanabe N, Sato N, Perera PY, Filkoski L, Tanaka T, et al. Antibody-mediated blockade of IL-15 reverses the autoimmune intestinal damage in transgenic mice that overexpress IL-15 in enterocytes. Proc Natl Acad Sci USA. 2009;106: 15849–15854. doi: 10.1073/pnas.0908834106 19805228 PMC2736142

[35] Seaman WE, Sleisenger M, Eriksson E, Koo GC. Depletion of natural killer cells in mice by monoclonal antibody to NK-1.1. Reduction in host defense against malignancy without loss of cellular or humoral immunity. J Immunol. 1987;138: 4539–4544. 3584981

[36] Carlyle JR, Mesci A, Ljutic B, Belanger S, Tai LH, Rousselle E, et al. Molecular and genetic basis for strain-dependent NK1.1 alloreactivity of mouse NK cells. J Immunol. 2006;176: 7511–7524. doi: 10.4049/jimmunol.176.12.7511 16751398

