# Supplementary figures and images for: Establishment of anti-asialo-GM1 rabbit monoclonal antibodies capable of reducing natural killer cell activity in mice
Source: PLoS One. 2023 Oct 9;18(10):e0292514. doi: 10.1371/journal.pone.0292514 (PMC10561865; doi:10.1371/journal.pone.0292514)

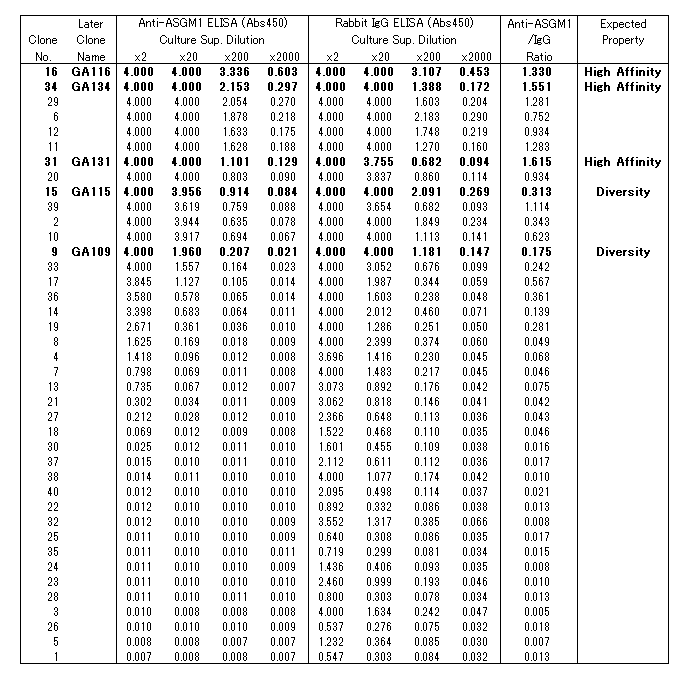

Supplement: S1 Table — Antibodies in serially diluted culture supernatants of HEK293 cells transfected with the antibody cDNA were measured using ELISA for anti-ASGM1 and whole-rabbit IgG. The results were reorganized according to the anti-ASGM1 titer order. The anti-ASGM1/rabbit IgG ratios were calculated from the absorbance at the lowest dilution within a linear range (Absorbance < 2.0 anti-ASGM1 ELISA). (TIF) [file pone.0292514.s001.tif]

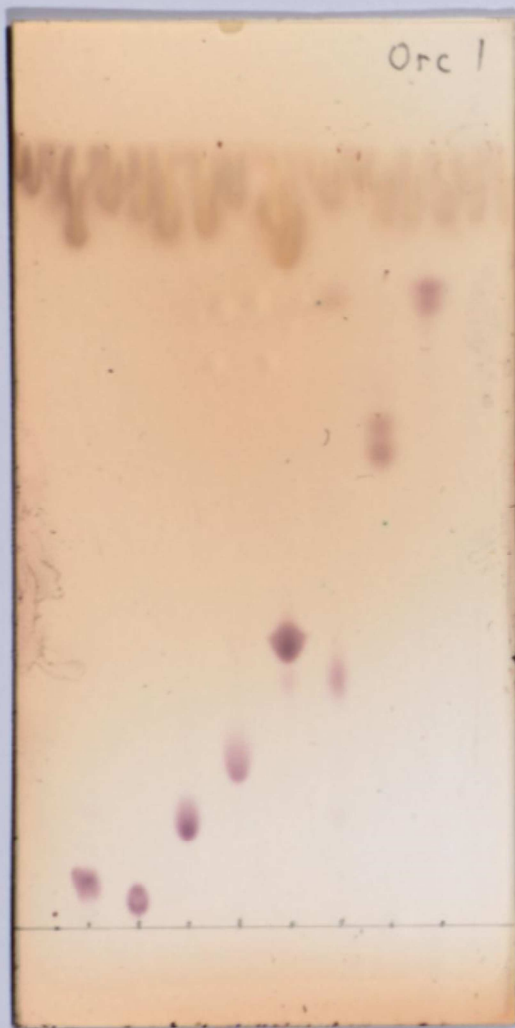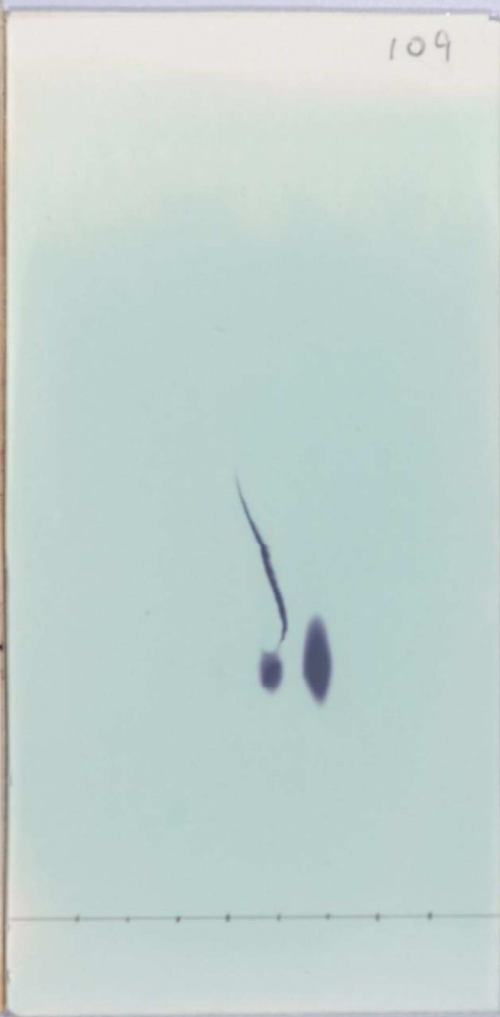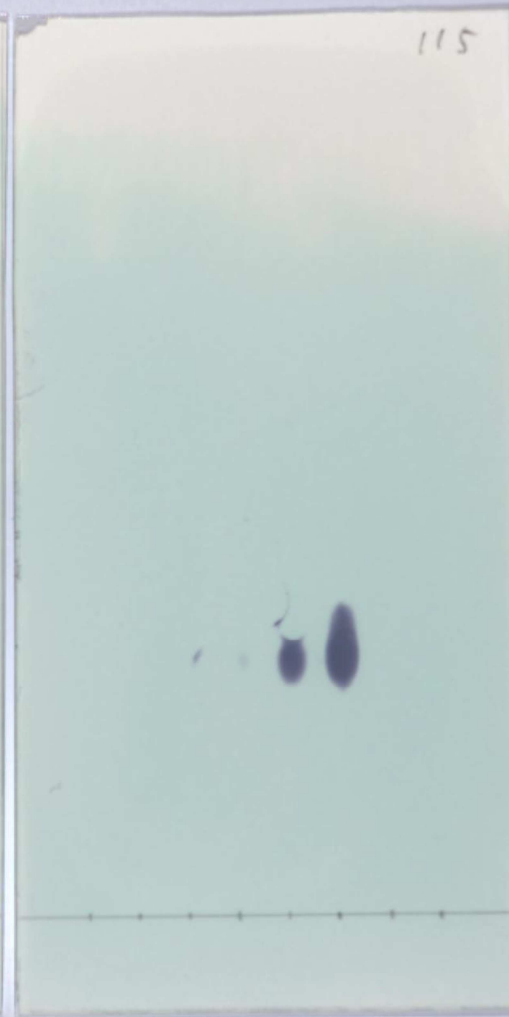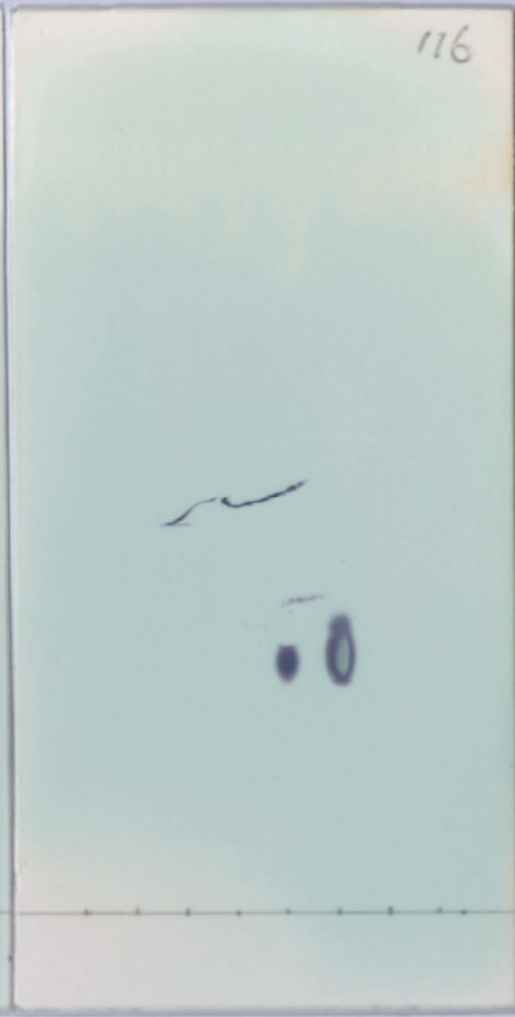

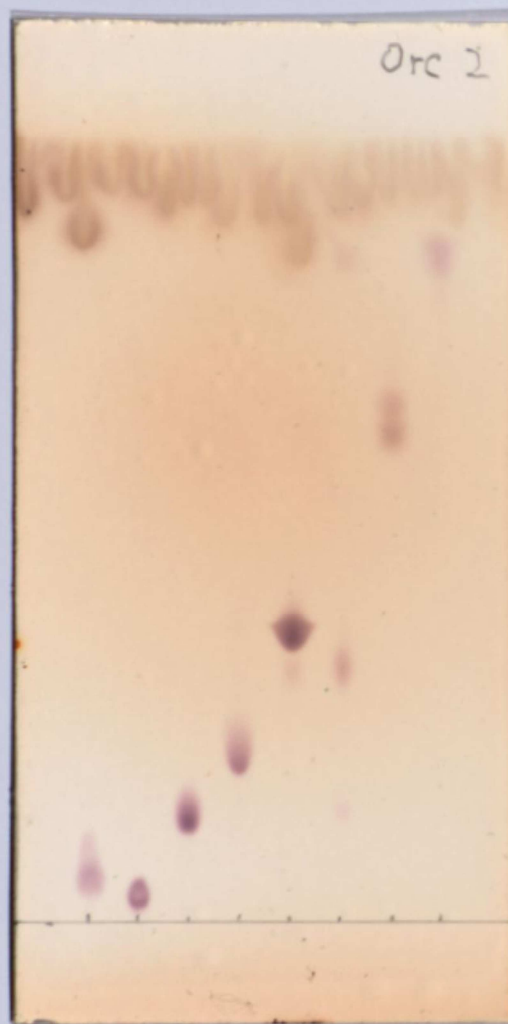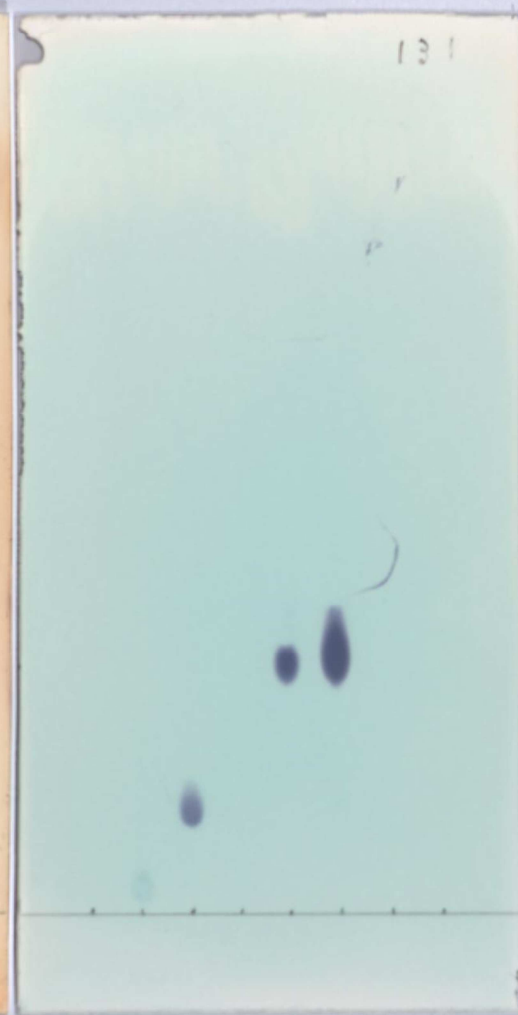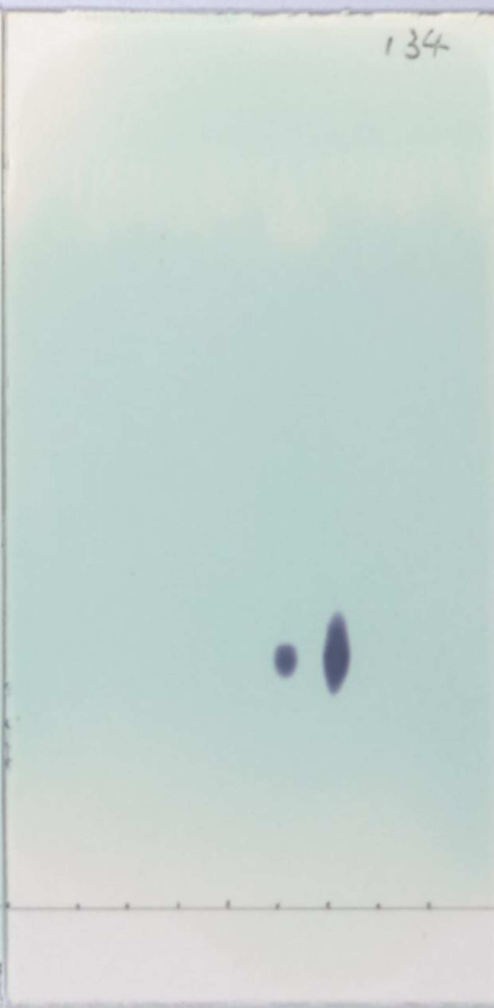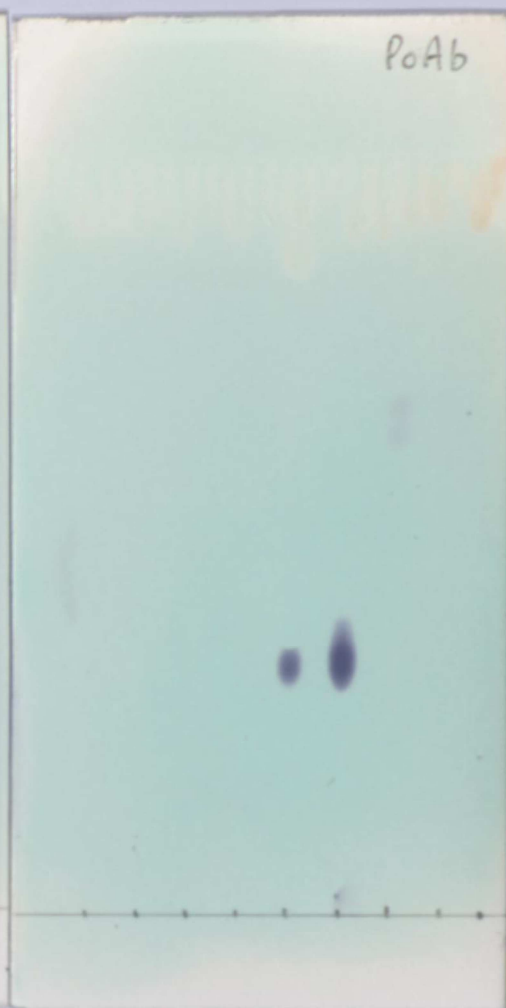

Supplement: S1 Raw images — (PDF) [file pone.0292514.s002.pdf]
